# Determinants of Cesarean Section Deliveries in Public Hospitals of Addis Ababa, Ethiopia, 2018/19: A Case-Control Study

**DOI:** 10.1155/2020/9018747

**Published:** 2020-04-20

**Authors:** Areaya Gebreegziabher Hailu, Tsegaye Kebede Fanta, Fissaha Tekulu Welay, Natnael Etsay Assefa, Surafel Aregawi Hadera, Gebrekiros Aregawi Gebremeskel, Hagos Weldeslassie Gebremedhin, Guesh Gebreayezgi Asefa

**Affiliations:** ^1^Department of Public Health, Gamby Medical and Business College, Bahir Dar, Ethiopia; ^2^Department of Midwifery, Adigrat University, Adigrat, Ethiopia; ^3^Department of Midwifery, Aksum University, Axum, Ethiopia; ^4^Department of Reproductive Health, Sante Medical College, Addis Ababa, Ethiopia; ^5^Department of Epidemiology and Biostatistics, School of Public Health, Akum University, Axum, Ethiopia

## Abstract

**Objective:**

The objective of this study was to assess the determinants of cesarean section deliveries in public hospitals of Addis Ababa, Ethiopia, 2019.

**Method:**

A hospital-based unmatched case-control study was conducted to study 780 (260 cases and 520 controls) women who delivered in public hospitals of Addis Ababa from August 22 to September 20, 2019. The cases were all mothers who delivered through caesarean section, and controls were all mothers who delivered vaginally in the same time in the study area. Data were collected from the randomly selected women and looking into their cards. Data were entered on EpiData 3.1 and exported to SPSS version 20 for cleaning and analyzing. Binary logistic regression and AOR with 95% CI were used to assess the determinants of caesarean section.

**Results:**

Majority of the study participants were in the age category 20–34 years. Nearly more than 1/3^rd^ of the participants (32.7% cases and 34.6% controls) have attended primary school. Most of the cases 217 (83.5%) and few of the controls 21 (4%) possess previous caesarean section. One hundred three (52.3%) of the cases and 329 (63.6%) controls were multi-parous. Previous caesarean delivery (AOR = 6.93, 95% CI; (3.39, 14.16)), singleton pregnancy (AOR = 0.34, 95% CI; (0.12, 0.83)), birth weight less than 2500 gm (AOR = 0.29, 95% CI; (0.18, 0.92)), birth weight greater than 4000 gm (AOR = 16.15 (8.22, 31.74)), completely documented partograph (AOR = 0.13, 95% CI; (0.078, 0.23)), and pregnancy-induced hypertension (AOR = 2.44, 95% CI; (1.46, 4.08)) were significant determinants of caesarean delivery in this study.

**Conclusion:**

Previous caesarean section, number of delivery, birth weight, partograph documentation, and pregnancy-induced hypertension had significant association with caesarean section delivery in this study.

## 1. Introduction

The proportion of caesarean section (CS) to the total births is considered as one of the important indicators of emergency obstetric care [1]. Currently, the WHO states that CS has paramount importance on reducing maternal and perinatal mortality and morbidity, provided that there is justified medical indication. However, in the absence of clear medical justification, CS has no medical benefit, rather it is associated with short- and long-term health risks as compared with vaginal delivery [2].

CS rates have been rising progressively worldwide with a wide variation between countries and regions. It is globally known that CS delivery can present several risks than a vaginal birth even if it has become nowadays one of obstetric intensive cares regarding its benefits for mother and baby [3].

Many studies indicated medical and nonmedical factors that are likely to be associated with the rising rate of CS which include social and obstetric maternal factors (age, educational status, income, preference, height, weight, parity, premature rupture of the amniotic fluid membrane, and multiple pregnancy), fetal factors (macrosomia, breech presentation, etc.), and indications (cephalopelvic disproportion, hypertensive disorders of pregnancy, antepartum hemorrhage, previous caesarean section, and fetal distress) [4–10].

Though the rate of CS is increasing in a normal pregnancy, CS has eight-fold higher maternal mortality and 8–12 times higher morbidity than vaginal delivery [11, 12]. Nowadays, the CS rate has become more prevalent without a clear medical justification though it is associated with an increased morbidity and mortality of mothers and children [13–17].

In 2015, the WHO suggested CS can save the life of the mother and infant as well only when it is medically justified [2]. The Ethiopian Demographic Health Survey (EDHS) 2016 reported over utilization of CS rates in Addis Ababa (21.4%) [18]. However, there is uncertainty if the determinants leading to CS delivery are up to the WHO recommendation. Thus, this study is intended to determine what factors are really predisposing mothers to CS delivery. Moreover, this study would help to have an extensive and up-to-date picture on the problem.

## 2. Main Text

### 2.1. Methods

A quantitative facility-based unmatched case-control study design was applied to study a total of 780 participant mothers from August 22 to September 20, 2019. The mothers were interviewed after they gave birth either by spontaneous vaginal delivery or caesarean section. Mothers who delivered a baby after fetal viability (28 weeks) were included, while those who were admitted to the postnatal ward after home delivery were excluded from this study. The sample size was calculated using Epi Info version 3.5.1 statistical software using the double population formula for the unmatched case-control study. Hence, considering the previous exposure of macrosomia baby (*p*1=52% for cases and *p*2=1.6%) and 10% nonresponse rate, the calculated value was 260 for cases and 520 for controls. Finally, the total number of samples was 780 mothers. The study was conducted on six public hospitals administered by Addis Ababa Health Bureau. Thus, the sample size was allocated proportionally based on the average delivery load in the past three months (February, March, and April). Cases and controls were selected exclusively using the systematic random sampling technique. The data were collected using a questionnaire and checklist. The questionnaire was structured, pretested, translated, and adapted from previous articles. Medical records of parturient mothers were used to extract information such as partograph, gestational age at delivery, fetal presentation, indication of CS, and birth weight of the fetus, while the information of the other variables were primary data taken from interview of postnatal mothers.

The data for this study were collected after ethical clearance was obtained from Gamby Medical and Business College and Addis Ababa public health research and emergency management care process. The participants were informed about the objective clearly and were also informed that the information they provided is only used for research purposes and kept confidential. Finally, the collected data were entered on EpiData3.1 and exported to SPSS version 20 for analysis. The variables with *p* < 0.25 on bivariate logistic regression were taken to multivariable logistic regression to control possible confounding factors. Finally, adjusted odds ratio with 95% confidence interval was used to measure strength of association between the predictors and occurrence of CS. Statistical significance was declared at *p* < 0.05.

Cases are mothers who delivered by caesarean section, whereas controls are those who delivered vaginally.

In this study, complete and partial partograph documentation is defined as if all the component of partograph was correctly filled and some components were missed, respectively, at the time of reviewing mother's card. Meanwhile, partograph is reported as undocumented if and only if the labor was not followed by Partograph at all.

Prolonged premature rupture of membrane is defined as duration of rupture of the membrane greater than 12 hours before the onset of labor [19].

Duration of labor: the duration of labor in this study is measured from the onset of true labor including the latent phase of first stage of labor to third stage of labor.

## 3. Result

### 3.1. Socioeconomic and Demographic Characteristics

The study recruited 780 mothers (260 cases and 520 controls) making a response rate of 100%. Majority of the participants (82.3% cases and 86% controls) were in the age category of 20–34 years. Nearly one third of enrolled mothers (32.7% cases and 34.6% controls) have attended primary school (Table 1).

### 3.2. Obstetric Characteristics of the Participants

More than half of cases and nearly 2/3^rd^ of controls were multi-parous. Twenty-seven (10.4%) of cases and fifty (9.6%) of controls had previous stillbirth. Eighty-nine (34.2%) of cases and fifty-two (10%) of controls had faced fetal distress during labor. Most (83.5%) of the cases and 4% of controls had previous CS delivery (Table 2).

**Figure 1:**
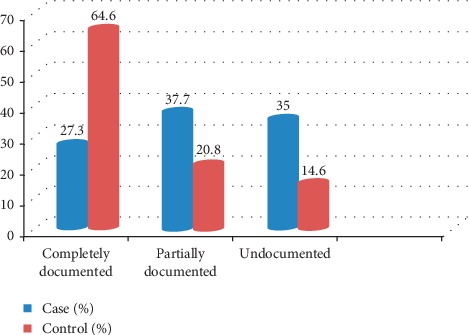
Bar graph showing status of partograph documentation for cases and controls among women who delivered in public hospitals of Addis Ababa, Ethiopia, 2019.

Fully documented partograph was higher in controls than cases, whereas more proportion of partially and not documented partograph was higher in cases than controls (Figure 1).

In Table 3, the Robson classification is used to compare the risk of delivering via CS among the ten groups of the delivering women. In this study, 2/3^rd^ (67.4%) of group five and more than half of group eight (54.5%) and group two (51.5%) of the Robson classification were delivered through CS delivery (Table 3).

### 3.3. Determinants of Caesarean Section Deliveries

Women who had previous CS and had completely documented partograph (AOR = 6.93, 95% CI; (3.39, 14.16) and (AOR = 0.13, 95% CI; (0.078, 0.23), respectively, were more likely to undergo CS than their counter parts (Table 4).

## 4. Discussion

CS is a life-saving procedure. Thus, the procedure should not be done without clear medical indication. In this study, mothers who underwent previous CS were 6.93 times more likely to deliver by CS for the consecutive birth compared with those who gave birth via vaginal delivery. This finding is consistent with the studies conducted in Bahir Dar, Harar, Addis Ababa, Mekelle, and Dessie [20–24]. This might be due to the fear of uterine rupture associated with waiting longer time.

Pregnancy-induced hypertension during labor was found risky for CS delivery. Hence, it is similar with the study conducted in Bukavu Provincial Hospital in Democratic Republic of Congo [25]. The possible reason could be long duration of vaginal delivery in case of pregnancy-induced hypertension.

In this study, neonatal birth weight ≥4000 gm was more likely to deliver through CS than birth weight of 2500–4000 gm. Perhaps, it is in line with the study done in eastern Ethiopia; Felege Hiwot Hospital, Oman, and DR Congo [20, 25, 26]. This is because macrosomia babies would face birth difficulty if vaginal delivery is allowed.

This study showed that completely filled partograph was 87% less likely to go to CS room than undocumented partograph. Studies conducted in Adigrat and Mekelle agreed with this study [23, 27]. This might be due to close monitoring of partograph decreasing the likelihood of CS rate.

Furthermore, singleton pregnancy was found as a protective factor for caesarian delivery.

### 4.1. Limitations of the Study

- Some very important factors like maternal height and body mass index (BMI) were not included as they are not routinely registered on medical cards at the setups where the research was conducted
- Scare to add qualitative part would not have given a chance for women to discuss more information about the decision-making process (attitude and perception of physicians and family)

## 5. Conclusion

In this study, only the obstetric characteristics were significant. Previous CS, number of delivery, birth weight, partograph documentation, and pregnancy-induced hypertension had significant association with CS delivery. Future studies need to examine the attitude of service providers and their influence on the growing CS delivery rate.

## Figures and Tables

**Table 1:** Socioeconomic and demographic characteristics of cases and controls in public hospitals of Addis Ababa, Ethiopia, 2019.

Variable	Category	Case	Control
		No (%)	No (%)
Age (years)	15–19	3 (1.2)	17 (3.2)
	20–34	214 (82.3)	447 (86)
	≥35	43 (16.5)	56 (10.8)

Marital status	Married	242 (93.1)	454 (87.2)
	Single	14 (5.4)	45 (8.7)
	Divorced	3 (1.1)	17 (3.3)
	Widowed	1 (0.4)	4 (0.8)

Educational status	Not able to read and write	43 (16.5)	78 (15)
	Grade 1–8	85 (32.7)	180 (34.6)
	Grade 9–12	66 (25.4)	130 (25)
	College and above	66 (25.4)	132 (25.4)

Occupational status	House wife	118 (45.4)	229 (44)
	Merchant	44 (16.9)	77 (14.8)
	Government employed	48 (18.5)	89 (17.1)
	Private employed	44 (16.9)	108 (20.8)
	Student	6 (2.3)	17 (3.3)

Monthly income	≤1000	37 (14.2)	82 (15.8)
	1001–2500	54 (20.8)	131 (25.2)
	2501–3999	43 (16.5)	132 (25.4)
	≥4000	126 (48.5)	175 (33.6)

**Table 2:** Obstetric characteristics of the participants of the study in public hospitals of Addis Ababa, Ethiopia, 2019.

Variable	Category	Case	Control
		No (%)	No (%)
Parity	Primipara	114 (38.8)	180 (61.2)
	Multipara with no previous CS	103 (24.4)	319 (75.6)
	Multipara with previous CS	43 (67.2)	21 (32.8)

Previous stillbirth	Yes	27 (10.4)	50 (9.6)
	No	233 (89.6)	470 (90.4)

Previous abortion	Yes	84 (32.3)	118 (22.7)
	No	176 (67.7)	402 (77.3)

Labor onset	Spontaneous	154 (59.2)	372 (71.5)
	Induced	106 (40.8)	148 (28.5)

ANC follow-up	Yes	246 (94.6)	502 (96.5)
	No	14 (5.4)	18 (3.5)

Number of ANC visit	1^st^ visit	9 (3.7)	7 (1.4)
	2^nd^ visit	21 (8.5)	21 (4.2)
	3^rd^ visit	44 (17.9)	86 (17.1)
	4^th^ visit	172 (69.9)	388 (77.3)

Emergency referral in	Yes	101 (38.8)	153 (29.4)
	No	159 (61.2)	367 (70.6)

Twin pregnancy	Yes	12 (4.6)	10 (1.9)
	No	248 (95.4)	510 (98.1)

Gestational age (weeks)	<37	8 (3.1)	41 (7.9)
	37–42	239 (91.9)	468 (90)
	>42	13 (5)	11 (2.1)

Fetal presentation at delivery	Cephalic	217 (83.5)	466 (89.6)
	Breech	36 (13.8)	47 (9.1)
	Transverse/oblique	7 (2.7)	7 (1.3)

Prolonged premature rupture of membrane	Yes	78 (30)	132 (25.4)
	No	182 (70)	388 (74.6)

Neonatal birth weight (gm)	<2500	28 (10.8)	126 (24.2)
	2500 to 4000	188 (72.3)	386 (74.2)
	>4000	44 (16.9)	8 (1.6)

Duration of labor (hours)	≤24	212 (81.5)	482 (92.7)
	>24	48 (18.5)	38 (7.3)

Previous CS	Yes	217 (83.5)	21 (4)
	No	43 (16.5)	499 (96)

Fetal distress	Yes	89 (34.2)	52 (10)
	No	171 (65.8)	468 (90)

APH	Yes	14 (5.4)	4 (0.8)
	No	246 (94.6)	516 (99.2)

PIH	Yes	69 (26.5)	68 (13.1)
	No	191 (73.5)	452 (86.9)

**Table 3:** Robson classification and risk of CS of the participants of the study in public hospitals of Addis Ababa, Ethiopia, 2019.

					Case (%)	Control (%)
Robson classification			Group 1	Nulliparous with single cephalic pregnancy, ≥37 weeks in spontaneous labor	34 (23.9)	108 (76.1)
			Group 2	Nulliparous with single cephalic pregnancy, ≥37 weeks who either had induced labor or CS delivery before labor	56 (51.5)	32 (48.5)
			Group 3	Multi-parous without previous uterine scar, single cephalic pregnancy, ≥37 weeks in spontaneous labor	51 (22.0)	181 (78.0)
			Group 4	Multi-parous without previous uterine scar, with single cephalic pregnancy, ≥37 weeks who either had induced labor or CS delivery before labor	23 (22.8)	78 (77.2)
			Group 5	All multi-parous with at least on previous CS, with single cephalic pregnancy, ≥37 weeks	31 (67.4)	15 (32.6)
			Group 6	All nulliparous with single breech pregnancy	10 (45.5)	12 (54.5)
			Group 7	All multi-parous with single breech pregnancy including previous uterine scars	25 (42.4)	34 (57.6)
			Group 8	All multiple pregnancy including previous uterine scars	12 (54.5)	10 (45.5)
			Group 9	All single pregnancy with transverse or oblique lie including women with previous uterine scars	0	0
			Group 10	All single cephalic pregnancy ≤37 weeks including women with previous uterine scars	5 (11.4)	39 (88.6)

**Table 4:** Logistic regression analysis results for determinants of CS delivery in public hospitals of Addis Ababa, Ethiopia, 2019.

Variable	Category	CS	VD	COR (95%)	AOR (95%)
		No (%)	No (%)		
Age (years)	15–19	3 (1.2)	17 (3.2)	0.230 (0.063, 0.83)	0.206 (0.038, 1.10)
	20–34	214 (82.3)	447 (86)	0.623 (0.40, 0.95)	0.69 (0.47, 1.13)
	≥35	43 (16.5)	56 (10.8)	1	1

Occupational status	House wife	118 (45.4)	229 (44)	1.265 (0.83, 1.91)	1.144 (0.63, 2.074)
	Merchant	44 (16.9)	77 (14.8)	1.403 (0.84, 2.34)	1.476 (0.73, 2.98)
	Government employed	48 (18.5)	89 (17.1)	1.324 (0.67, 1.54)	1.35 (0.68, 2.67)
	Private employed	44 (16.9)	108 (20.8)	1	1

Previous abortion	Yes	84 (32.3)	118 22.7)	1.623 (1.17, 2.26)	0.94 (0.592, 1.49)
	No	176 (67.7)	402 (77.3)	1	1

ANC follow-up	Yes	246 (94.6)	502 (96.5)	0.63 (0.31, 1.29)	1.93 (0.316, 11.82)
	No	14 (5.4)	18 (3.5)	1	1

Previous CS	Yes	43 (16.5)	21 (4)	4.71 (2.73, 8.13)	6.93 (3.39, 14.16)^*∗*^
	No	217 (83.5)	499 (96)	1	1

Emergency referral	Yes	101 (38.8)	153 (29.4)	1.52 (1.12, 2.08)	1.02 (0.66, 1.58)
	No	159 (61.2)	367 (70.6)	1	1

Pregnancy-induced hypertension	Yes	69 (26.5)	68 (13.1)	2.40 (1.65, 3.49)	2.44 (1.46, 4.08)^*∗*^
	No	191 (73.5)	452 (86.9)	1	1

Twin delivery	No	248 (95.4)	510 (98.1)	0.40 (0.17, 0.95)	0.34 (0.12, 0.83)^*∗*^
	Yes	12 (4.6)	10 (1.90)	1	1

Prolonged premature rupture of membrane	Yes	78 (30)	132 (25.4)	1.26 (0.91, 1.75)	1.39 (0.88, 2.20)
	No	182 (70)	388 (74.6)	1	1

Birth weight (gm)	<2500	28 (10.8)	126 (24.2)	0.46 (0.29, 0.72)	0.29 (0.18, 0.92)^*∗*^
	2500–4000	188 (72.3)	386 (74.2)	1	1
	>4000	44 (16.9)	8 (1.6)	11.3 (5.2, 24.5)	16.15 (8.22, 31.74)^*∗*^

Partograph documentation	Completely documented	71 (27.3)	336 (64.6)	0.176 (0.12, 0.26)	0.13 (0.078, 0.23)^*∗*^
	Partially documented	98 (37.7)	108 (20.8)	0.758 (0.50, 1.14)	0.64 (0.37, 1.10)
	Undocumented	91 (35)	76 (14.6)		1

COR = crude odds ratio, AOR = adjusted odds ratio ^*∗*^Statistically significant at *p* < 0.05.

## Data Availability

The datasets during and/or analyzed on the current study are available from the corresponding author on reasonable request.
